# Pulsed-field ablation—are we ready for fast and furious atrial tachycardia ablation?

**DOI:** 10.1007/s10840-023-01510-2

**Published:** 2023-02-20

**Authors:** Christian-Hendrik Heeger, Roland Richard Tilz

**Affiliations:** 1 Deparment of Rhythmology, University Heart Center Lübeck, University Hospital Schleswig-Holstein, Lübeck, Germany; 2 German Center for Cardiovascular Research (DZHK), Partner Site Hamburg/Kiel/Lübeck, Lübeck, Germany; 3 LANS Cardio, Stephansplatz 5, 20354 Hamburg, Germany

Pulsed-field ablation (PFA) is a novel ablation technology with promising safety and efficacy advantages [1]. Contrary to latest thermal ablation energy sources like radiofrequency, laser light, and cryotherapy, PFA is a non-thermal ablation modality. Ablation of the target tissue is therefore not performed by thermal injury of the cells but by exposition to repetitive and rapid high-voltage electrical fields. This exposition results in destabilization of the cell membranes followed by cell death. The different resistance of different cell types (e.g., smooth muscle cells, nervous system cells, myocardial cells) offers the opportunity of targeting only myocardial cells while sparing non-target cells which is minimizing collateral damage [2]. The FARAWAVE (FARAPULSE, Boston Scientific, Marlborough, Massachusetts) catheter received the CE mark in 2021 and was the first available PFA ablation catheter. Although its multi-electrode and penta spline shape was designed for pulmonary vein isolation, latest research focused also on treatment of further arrhythmias [3, 4]. In this issue of the *Journal of Interventional Cardiac Electrophysiology*, Kueffer et al. present their first experience of the FARAWAVE catheter for treatment of left atrial (LA) reentry tachycardia in combination with a 3D mapping system. In 22 patients, a total of 27 common LA tachycardias have been treated by PFA utilizing the FARAWAVE catheter. Deployment of lines was performed by consecutive applications on the target area utilizing the flower or basket shape according to the best possible anatomic position. A total of 20 roof lines have been deployed, and bidirectional block was achieved in 100%. It is remarkable to state that additional radiofrequency ablation was necessary to block 15% of anterior lines (in total *n* = 13) and 50% of mitral lines (in total *n* = 6). Furthermore, despite additional radiofrequency ablation, bidirectional block was achieved in 92% of anterior lines and 83% of mitral isthmus lines. The colleagues observed no acute periprocedural complications utilizing this approach, and especially no clinically relevant coronary artery spasm has been observed. One hundred percent success rate of roof line deployment and isolation of the posterior wall is driven (1) by the catheter design in the flower position of the FARAWAVE catheter and (2) by the relatively thin tissue of the left atrial posterior wall [5]. The lesion formation of the FARAWAVE catheter seems to create durable transmural lesions in this area. Due to the specific characteristics of PFA with sparing of non-myocardial tissue, this novel energy source might offer an ideal tool for posterior wall isolation and roofline deployment on the one hand with simultaneously prevention of esophageal injuries.

The LA anterior wall and the mitral isthmus area are providing thicker myocardial tissue compared to the left atrial posterior wall. The lower success rates of line deployment in those areas might be driven by this fact. Furthermore, the catheter design seems to offer not the ideal shape for line deployment at the LA anterior wall and mitral isthmus area. Additionally, the depth of PFA-driven lesion formation seems to be debatable in those areas. Although no clinically relevant coronary artery spasm has been reported by Kueffer et al., recent findings, e.g., by Gunawardene et al. [6] and Reddy et al. [7], showed coronary artery spasm occurrence after limited PFA applications. Therefore, PFA deployment on the mitral isthmus area which closely related to the right circumflex artery should be avoided with the recent available PFA catheter design.

In summary deployment of common left atrial lines with the FARAPULSE system was safe and feasible in most cases. However, additional radiofrequency was necessary to achieve bidirectional block at the anterior wall and mitral isthmus area. A focal PFA catheter design may be more suitable for deployment of those lines. Due to latest reports on coronary artery spasm, a utilization of the FARAPULSE system on the mitral isthmus area cannot be recommended and needs to be intensively studied.
